# Transcriptome Analysis of Peripheral Blood Mononuclear Cells in Pulmonary Sarcoidosis

**DOI:** 10.3389/fmed.2022.822094

**Published:** 2022-01-24

**Authors:** Keiichiro Yoshioka, Hironori Sato, Takeshi Kawasaki, Daisuke Ishii, Takuro Imamoto, Mitsuhiro Abe, Yoshinori Hasegawa, Osamu Ohara, Koichiro Tatsumi, Takuji Suzuki

**Affiliations:** ^1^Department of Respirology, Graduate School of Medicine, Chiba University, Chiba, Japan; ^2^Department of Applied Genomics, Kazusa DNA Research Institute, Chiba, Japan; ^3^Department of Pediatrics, Graduate School of Medicine, Chiba University, Chiba, Japan

**Keywords:** RNA sequencing, transcriptome analysis, pulmonary sarcoidosis, peripheral blood mononuclear cell, granuloma formation

## Abstract

**Background:**

Sarcoidosis is a granulomatous systemic disease of unknown etiology. Mononuclear cells such as macrophages or lymphocytes in lung tissue and hilar or mediastinal lymph nodes have been recognized to play an essential role in granuloma formation in pulmonary sarcoidosis. Peripheral blood mononuclear cells (PBMCs) consist of several immunocompetent cells and have been shown to play a mechanistic role in the pathogenesis of sarcoidosis. However, the genetic modifications that occur in bulk PBMCs of sarcoidosis remain to be elucidated.

**Purpose:**

This study aimed to explore the pathobiological markers of sarcoidosis in PBMCs by comparing the transcriptional signature of PBMCs from patients with pulmonary sarcoidosis with those of healthy controls by RNA sequencing.

**Methods:**

PBMC samples were collected from subjects with pulmonary sarcoidosis with no steroid/immunosuppressant drugs (*n* = 8) and healthy controls (*n* = 11) from August 2020 to April 2021, and RNA sequencing was performed with the PBMC samples.

**Results:**

Principal component analysis using RNA sequencing datasets comparing pulmonary sarcoidosis with healthy controls revealed that the two groups appeared to be differentiated, in which 270 differentially expressed genes were found in PBMCs between sarcoidosis and healthy controls. Enrichment analysis for gene ontology suggested that some biological processes related to the pathobiology of sarcoidosis, such as cellular response to interleukin (IL)-1 and IFN-γ, regulation of IL-6 production, IL-8 secretion, regulation of mononuclear cell migration, and response to lipopolysaccharide, were involved. Enrichment analysis of the KEGG pathway indicated the involvement of tumor necrosis factor (TNF), toll-like receptor signaling, IL-17 signaling pathways, phagosomes, and ribosomes. Most of the genes involved in TNF and IL-17 signaling pathways and phagosomes were upregulated, while most of the ribosome-related genes were downregulated.

**Conclusion:**

The present study demonstrated that bulk gene expression patterns in PBMCs were different between patients with pulmonary sarcoidosis and healthy controls. The changes in the gene expression pattern of PBMCs could reflect the existence of sarcoidosis lesions and influence granuloma formation in sarcoidosis. These new findings are important to strengthen our understanding of the etiology and pathobiology of sarcoidosis and indicate a potential therapeutic target for sarcoidosis.

## Introduction

Sarcoidosis is a systemic granulomatous disease of unknown etiology that can cause dysfunction in various organs. The development of sarcoidosis can be influenced by genetic factors, environmental stimulation, and foreign or self-antigens (1). Granuloma formation is initiated by the accumulation of antigen-presenting cells, such as activated monocytes/macrophages and CD4^+^ Th cells. Granulomas consist of monocyte-derived epithelioid and giant cells encircled by various immune-competent cells such as CD4 + helper T (Th) cells, CD8 T cells, fibroblasts, regulatory T cells, and B lymphocytes (2). A variety of agents can be phagocytosed by antigen-presenting cells of monocytes/macrophages, leading to the activation of immune cells including T cells. Activated CD4^+^ Th cells then differentiate into Th1 cells, secreting primarily interleukin (IL)-2 and IFN-γ (3). Th1 cells have been shown to play a key role in granuloma formation in sarcoidosis. Recently, IFN-γ-derived Th17.1 cells have also been suggested to be involved in sarcoidosis (4). In any case, mononuclear cells such as monocytes/macrophages or lymphocytes have been recognized to play a central mechanistic role in granuloma formation in sarcoidosis. However, the mechanisms and general epidemiology of sarcoidosis remain unclear.

Peripheral blood mononuclear cells (PBMCs) comprise immunocompetent cells, which are mainly classified into two cell types: monocytes and lymphocytes. Monocytes are defined as CD14^+^ cells in PBMCs (5), which can be further categorized into three populations with different functions by the combination of CD14 expression level and CD16 expression: CD14^++^CD16^−^ classical, CD14^++^CD16^+^intermediate, and CD14^+^CD16^+^non-classical monocytes. Lymphocytes can be mainly categorized into three groups: T, B, and natural killer cells, and each population is known to comprise cells with wide variation in functional roles (6, 7). As such, PBMCs, consisting of various cell types, are considered to play important roles in immune-related diseases, including sarcoidosis.

Omics is a recently developed analysis method that encompasses genomics, epigenomics, transcriptomics, proteomics, and metabolomics, and has already been widely used to understand polygenic and phenotypically diverse diseases. In particular, transcriptomics has also been applied to understand the pathogenesis of sarcoidosis at the levels of PBMCs, monocytes, regulatory T cells, and single cells of PBMCs, to explore specific genetic expressions of mRNAs or microRNAs that might explain disease progression and distinguish it from other diseases (8, 9).

A previous study showed that gene expression in PBMCs was different between sarcoidosis patients and healthy controls (10); however, some data included in the analysis were from sarcoidosis patients who received steroids and/or immunosuppressant drugs. Therefore, the transcriptome status may be largely affected by drugs that mask some notable differences between sarcoidosis patients and healthy controls. Taking this into consideration, it would be worth performing transcriptome analysis comparing healthy controls with sarcoidosis patients who do not take steroids or immunosuppressant drugs.

Therefore, this study aimed to explore the pathobiological significance of PBMCs in sarcoidosis by comparing the transcriptional signature of PBMCs from healthy controls with that from pulmonary sarcoidosis subjects not receiving steroids and/or immunosuppressant drugs.

## Materials and Methods

### Subjects

This study was approved by the Human Ethics Committee of Chiba University (protocol number 2083). PBMCs were collected from patients diagnosed with pulmonary sarcoidosis and from healthy controls between August 2020 and April 2021 at Chiba University Hospital. Patients with pulmonary sarcoidosis were diagnosed according to the recommendations of the ATS/ERS/WASOG statement (11), and all patients who had no histopathological confirmation of granulomas were excluded from the present study.

### Isolation of PBMC

Peripheral blood was collected using a BD Vacutainer CPT Cell Preparation Tube with sodium citrate according to the manufacturer's protocol (#362760) (Becton, Dickinson and Company, NJ, USA). Briefly, the tubes containing blood were centrifuged at 1,500 rpm at room temperature for 20 min. After centrifugation, the plasma was removed from the uppermost layer. The PBMC layer was transferred to 15 ml conical tubes, washed with PBS twice, followed by the addition of Isogen (Nippongene, Tokyo, Japan), and then stored at −80°C.

### Total RNA Extraction, mRNA Library Preparation, and 3'RNA-Seq

Total RNA was extracted from 1.0 to 2.0 × 10^6^ PBMCs, and each sample was transferred to 1.0 ml isogen reagent (Life Technologies, Carlsbad, CA, USA). The solution was vigorously vortexed and incubated at room temperature for 5 min. The solution was then centrifuged after adding chloroform, and the aqueous phase was carefully transferred to a new tube, following which 10 mg glycogen (Life Technologies) was added as a co-precipitant. RNA was precipitated by adding 600 μL of isopropyl alcohol. The RNA pellet was washed once with 75% ethanol and then dissolved in 10 μL RNase-free water. The concentration and quality of the RNA were verified using a Qubit fluorometer (Life Technologies) and an Agilent 2100 bioanalyzer, respectively. Purified total RNA (200 ng) was used for RNA library preparation, according to the instructions of the Quant Seq 3' mRNA-seq library preparation kit FWD for Illumina (Lexogen, Vienna, Austria). The RNA libraries were sequenced on an Illumina NextSeq 500 system with 75-nt-long reads.

### 3' RNA-Seq Data Analysis

RNA-seq count data were analyzed using the iDEP.92 (integrated differential expression and pathway analysis online tools: the details of analysis are described in the following website (http://bioinformatics.sdstate.edu/idep92/) and published paper (12). Briefly, expression matrix was pre-processed by filtering more than 0.5 counts per million per sample (pseudo count 4) and converted to Ensemble gene IDs, which are used internally to identify genes. These data were used for principal component analysis (PCA) and hierarchical clustering. RNA-seq count data after the filtration were normalized using EdgeR, an R/Bioconductor package connected to iDEP.92. In addition, DEGs from two group comparisons were obtained using the DESeq2 package with a threshold of false discovery rate (FDR) < 0.05, and fold-change > 2 or < 0.5. For enrichment analysis of the DEGs, gene set enrichment analysis (GSEA) was used to perform Gene Ontology (GO) analysis and Kyoto Encyclopedia of Genes and Genomes (KEGG) pathway analysis.

### Statistical Analysis

The age characteristics of the samples are expressed as means ± standard deviations. Student's *t*-test was used for age comparisons. We compared the samples for gender characteristics using Fisher's exact test. Statistical significance was set at *P* < 0.05.

## Results

### Differential Gene Expression and Pathway Analysis in PBMC

The present study included two groups: subjects comprising 8 patients with pulmonary sarcoidosis and 11 healthy controls. The subjects' demographics are shown in Table 1. Four patients with pulmonary sarcoidosis had a smoking history, and none were on immunosuppressive medication. All pulmonary sarcoidosis patients had lung involvement with chest radiograph stages 1, 2, or 3. There was a significant difference in age between patients with pulmonary sarcoidosis and healthy controls (*p* < 0.05).

**Table 1:** Characteristics of samples.

**Pulmonary sarcoidosis**					
**Samples**	**Male/female**	**Age**	**Stage**	**Organ involvements**	**Smoking history**
No.1	Female	76	3	Eye, heart	None
No.2	Male	56	1	Heart	None
No.3	Male	47	3	None	None
No.4	Female	68	3	Heart, liver, kidney	Yes
No.5	Female	59	2	None	Yes
No.6	Female	43	1	None	None
No.7	Male	62	1	None	Yes
No.8	Female	49	2	None	Yes
**Healthy controls**			
**Samples**	**Male/female**	**Age**	**Smoking history**
No.1	Male	42	None
No.2	Male	31	None
No.3	Female	34	None
No.4	Female	58	None
No.5	Female	45	None
No.6	Female	46	None
No.7	Female	48	None
No.8	Female	43	None
No.9	Male	66	None
No.10	Female	37	None
No.11	Female	40	None
**Pulmonary sarcoidosis vs. healthy controls**			
	**Pulmonary sarcoidosis**	**Healthy controls**	***P*** **-value**
Age	58.1 ± 11.5	44.5 ± 10.2	0.01^*^
Gender (male/female)	3/5	3/8	1.0^#^

* *Student's t-test*.

# *Fisher's exact test*.

We prepared RNA-seq libraries from mRNA isolated from the PBMCs of subjects with pulmonary sarcoidosis and healthy controls. The RNA integrity number values of all samples were >8, and 26,472 genes from mRNA were obtained. After additional quality control methods, 18,139 genes were retained as DEGs for further analyses. Next, the count data obtained by RNA-seq were processed and analyzed using iDEP.92.

**Figure 1:**
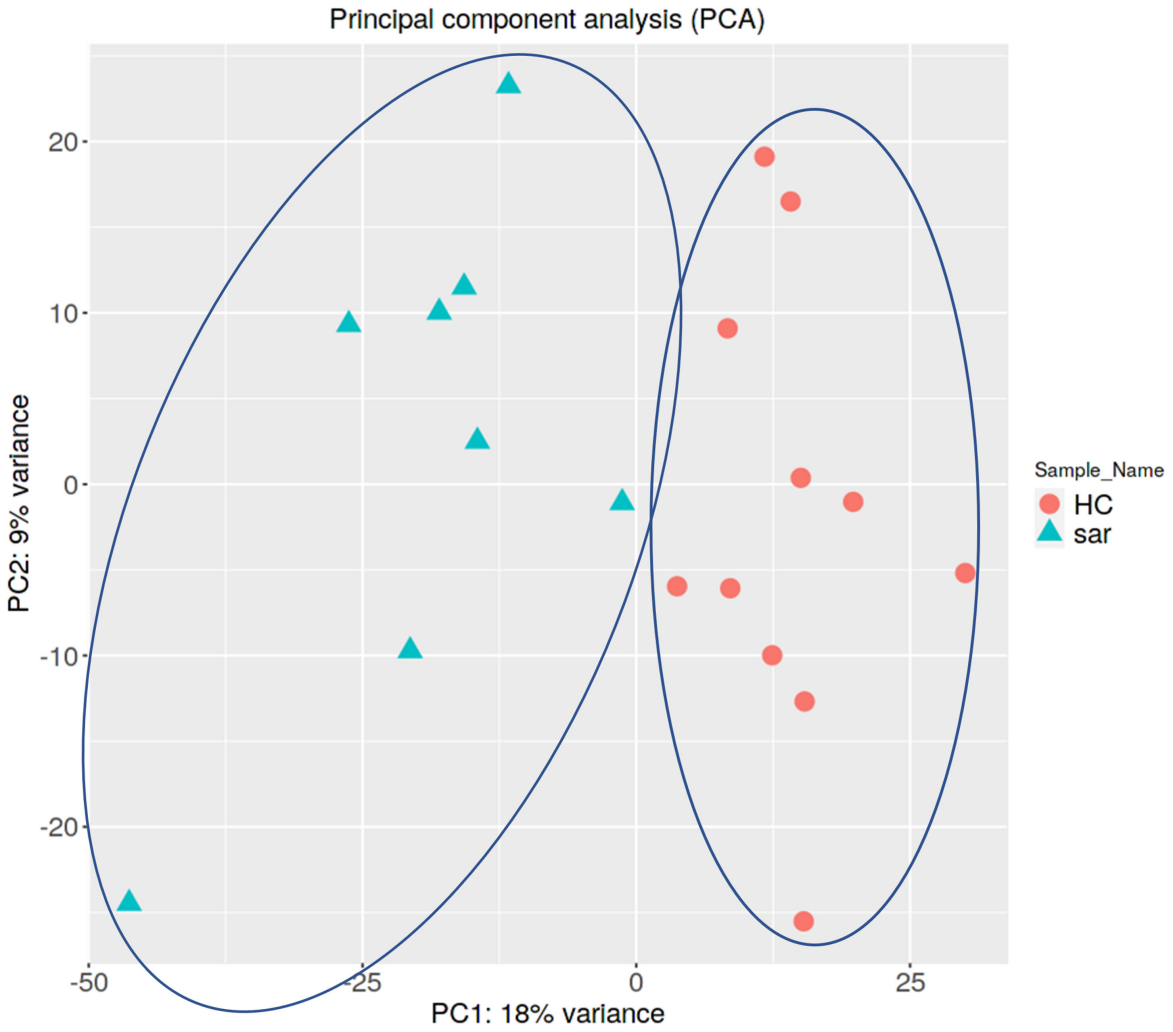
Principal component analysis (PCA). Principal component analysis (PCA) shows that two groups of pulmonary sarcoidosis and healthy controls are well-differentiated.

**Figure 2:**
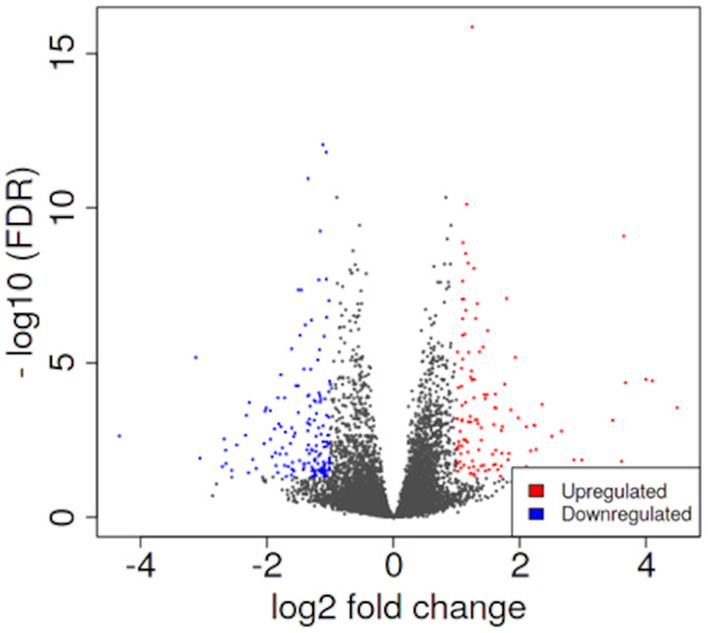
Volcano plotting of differentially expressed genes (DEGs). The distribution of log_2_-fold change and FDR expression level for the 18,139 genes are shown in volcano plotting. Dots highlighted in color are 270 DEGs between pulmonary sarcoidosis and healthy controls.

**Figure 3:**
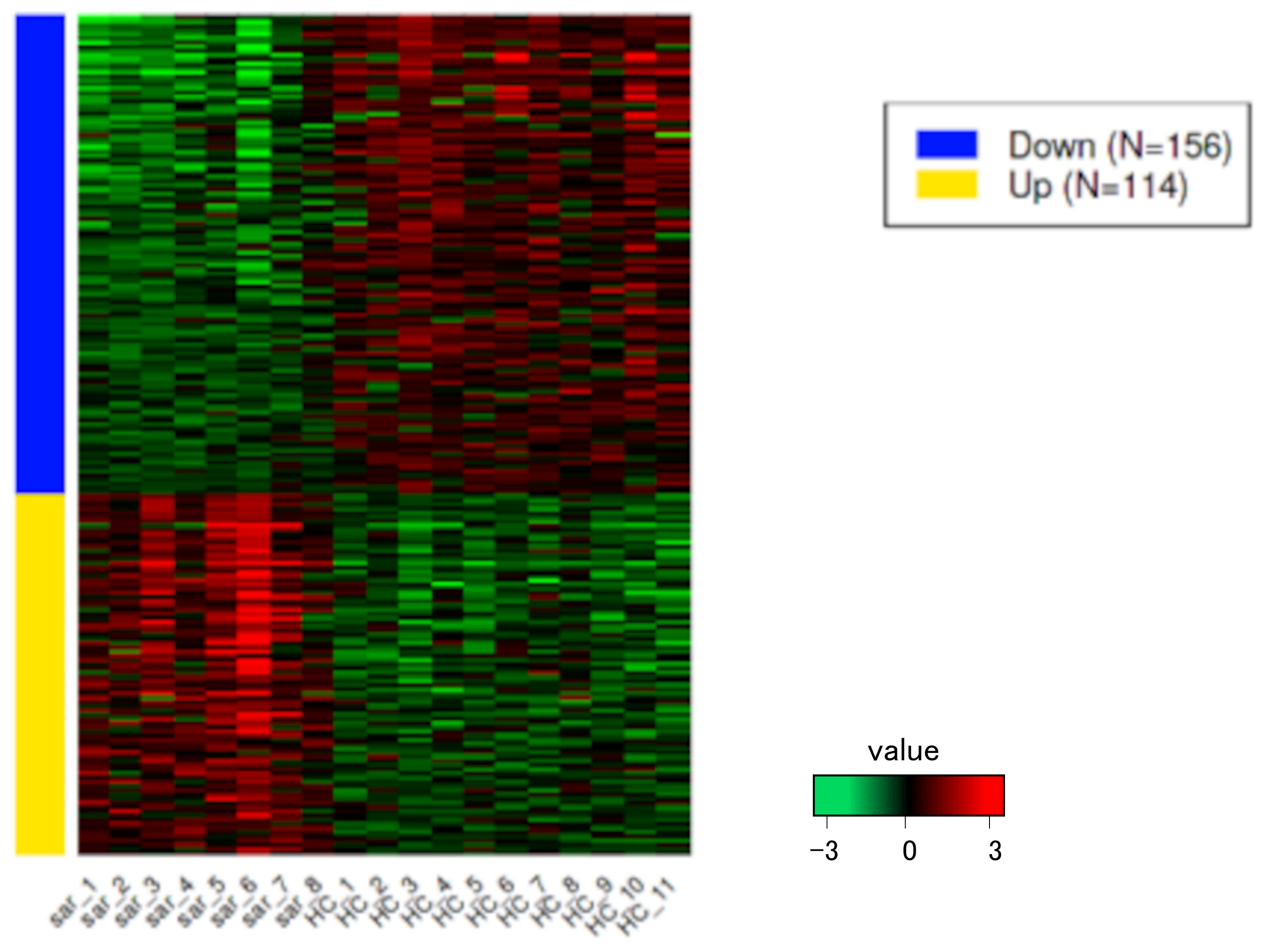
Heatmap of DEGs. The heatmap shows the DEGs between pulmonary sarcoidosis vs. healthy controls. Red bars represent high expression and green bars represent low expression.

PCA enables us to project samples into two-dimensional space (12). We performed PCA by comparing gene expression levels using count data between patients with pulmonary sarcoidosis and healthy controls. The results showed that the two groups appeared to be divided, as shown in Figure 1. Next, we compared the DEGs in PBMCs between patients with pulmonary sarcoidosis and healthy controls using DEseq2. Figure 2 shows the distribution of log_2_-fold change and FDR expression levels for the 18,139 genes expressed in these samples by volcano plotting with 270 DEGs (FDR < 0.05 and fold-change > 2 or < 0.5) highlighted in color. Figure 3 shows the heat map of the 270 DEGs, with 156 genes downregulated, and 114 genes upregulated in PBMCs of pulmonary sarcoidosis patients compared to healthy controls; the blue bar indicates genes that are upregulated in healthy controls, while the yellow bar indicates genes that are upregulated in patients with pulmonary sarcoidosis.

**Figure 4:**
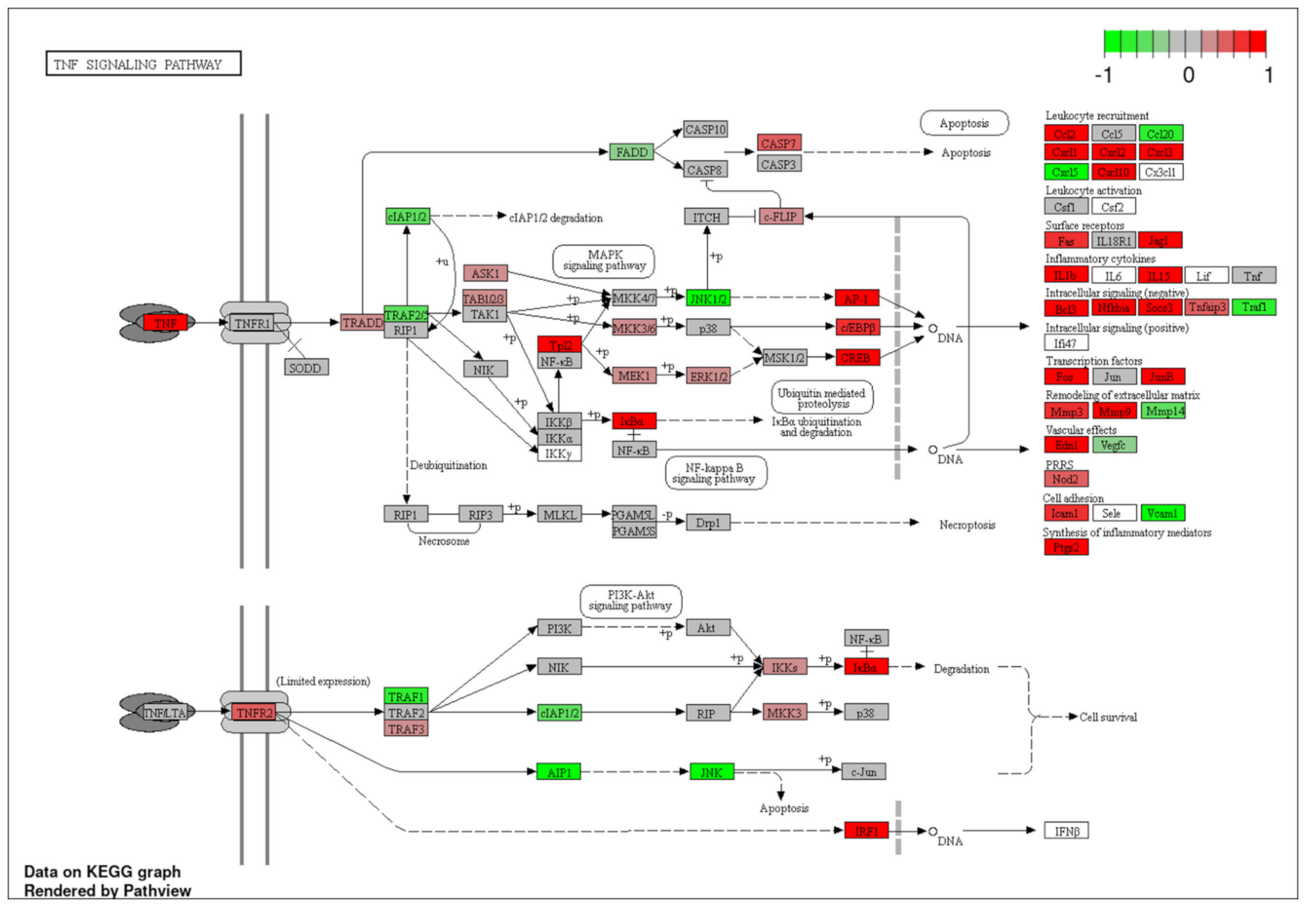
Genes involved in tumor necrosis factor (TNF) signaling pathway in pulmonary sarcoidosis peripheral blood mononuclear cells (PBMCs) by Kyoto Encyclopedia of Genes and Genomes (KEGG). A graphical illustration of KEGG pathway analysis of DEGs (log_2_-fold change with FDR < 0.05) related to TNF signaling pathway in pulmonary sarcoidosis PBMCs. The color intensity corresponds to the levels of upregulation (red) or downregulation (green) of the DEGs in pulmonary sarcoidosis PBMCs.

**Figure 5:**
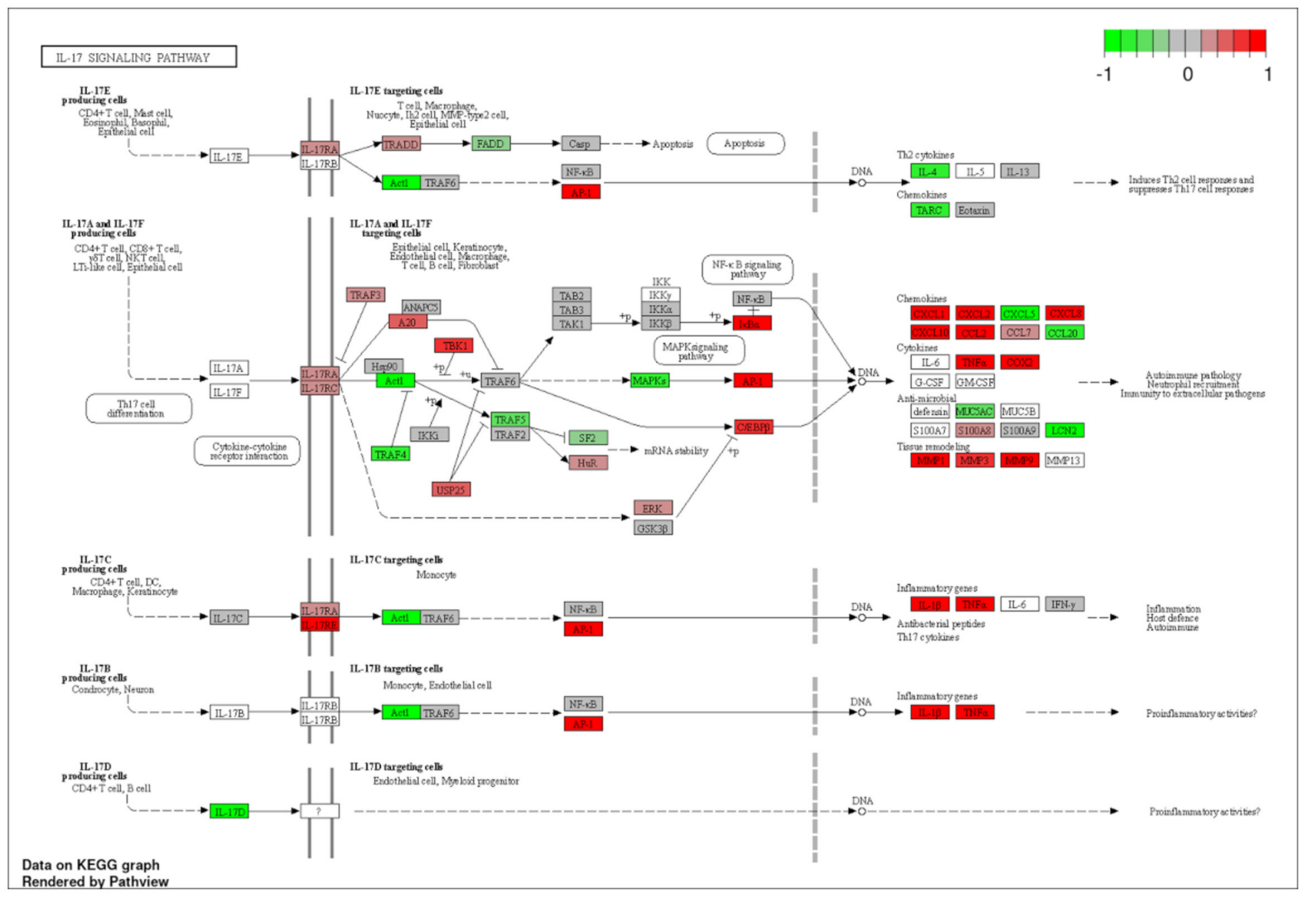
Genes involved in interleukin (IL)-17 signaling pathway in pulmonary sarcoidosis PBMCs by KEGG. A graphical illustration of KEGG pathway analysis of DEGs (log_2_-fold change with FDR < 0.05) related to the IL-17 signaling pathway in pulmonary sarcoidosis PBMCs.

**Figure 6:**
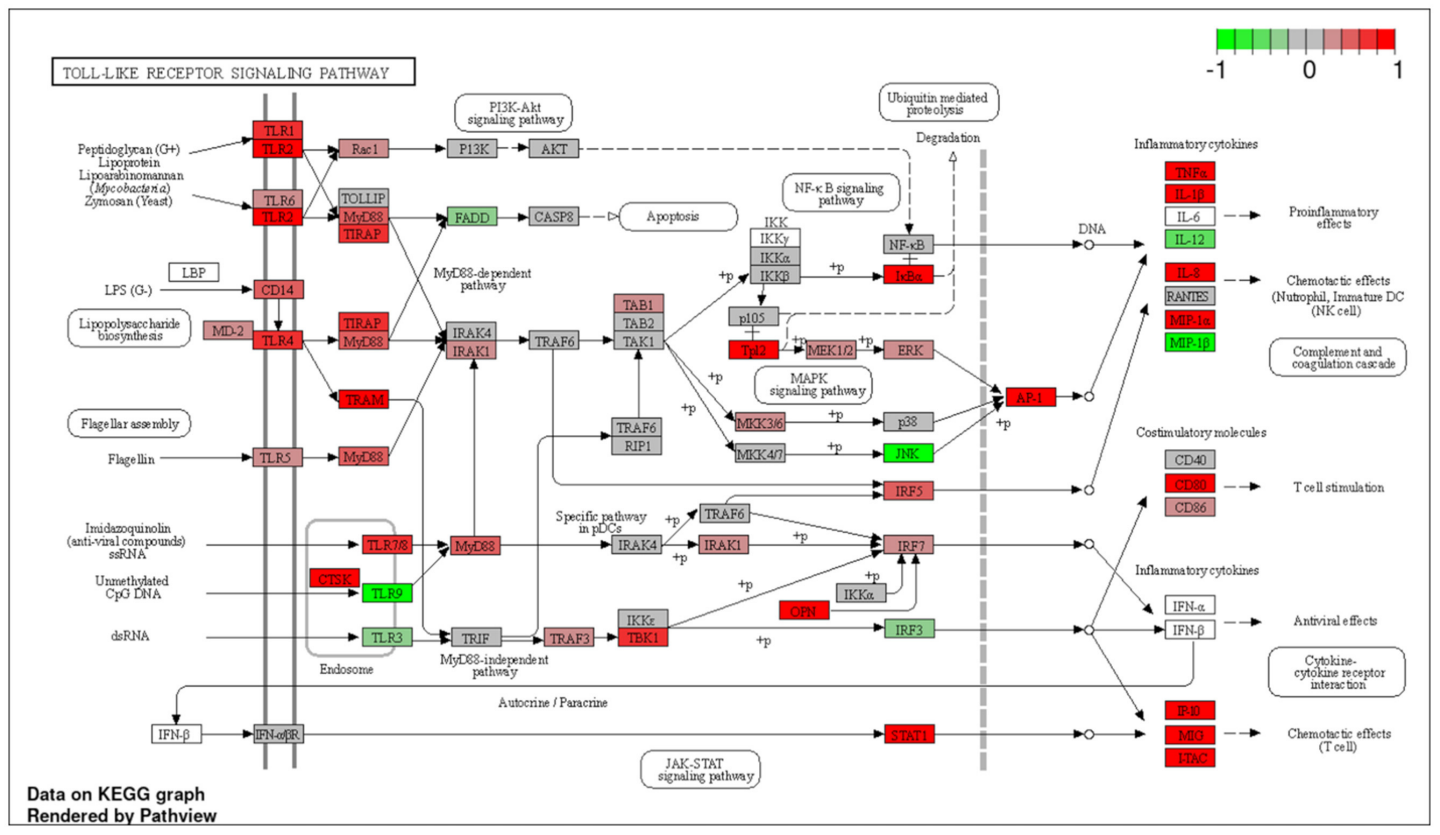
Genes involved in Toll-like receptor signaling pathway in pulmonary sarcoidosis PBMCs by KEGG. A graphical illustration of KEGG pathway analysis of DEGs (log_2_-fold change with FDR < 0.05) related to the Toll-like receptor signaling pathway in pulmonary sarcoidosis PBMCs.

**Figure 7:**
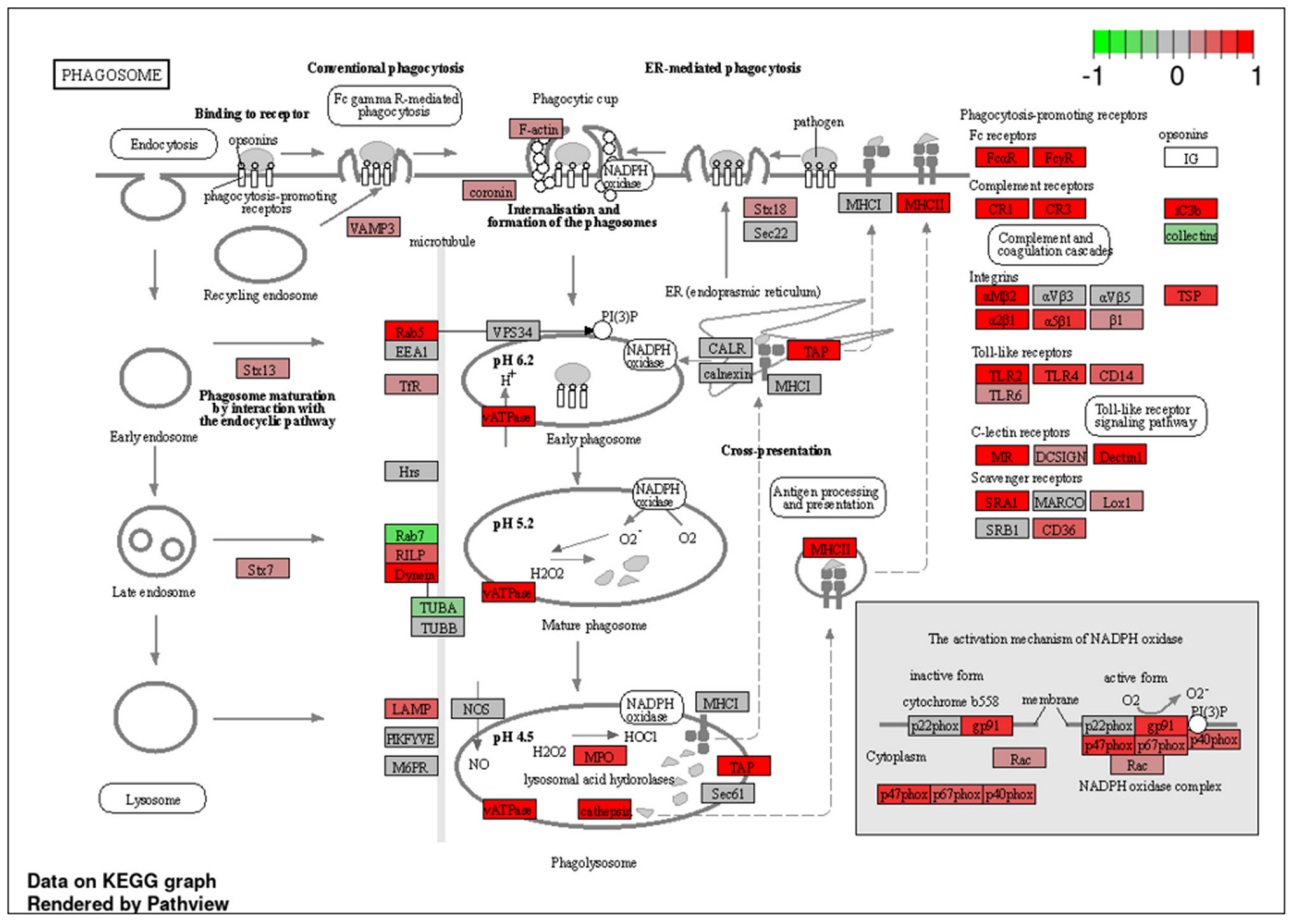
Genes involved in phagosome signaling pathway in pulmonary sarcoidosis PBMCs by KEGG. A graphical illustration of KEGG pathway analysis of DEGs (log_2_-fold change with FDR < 0.05) related to phagosome signaling pathway in pulmonary sarcoidosis PBMCs.

**Figure 8:**
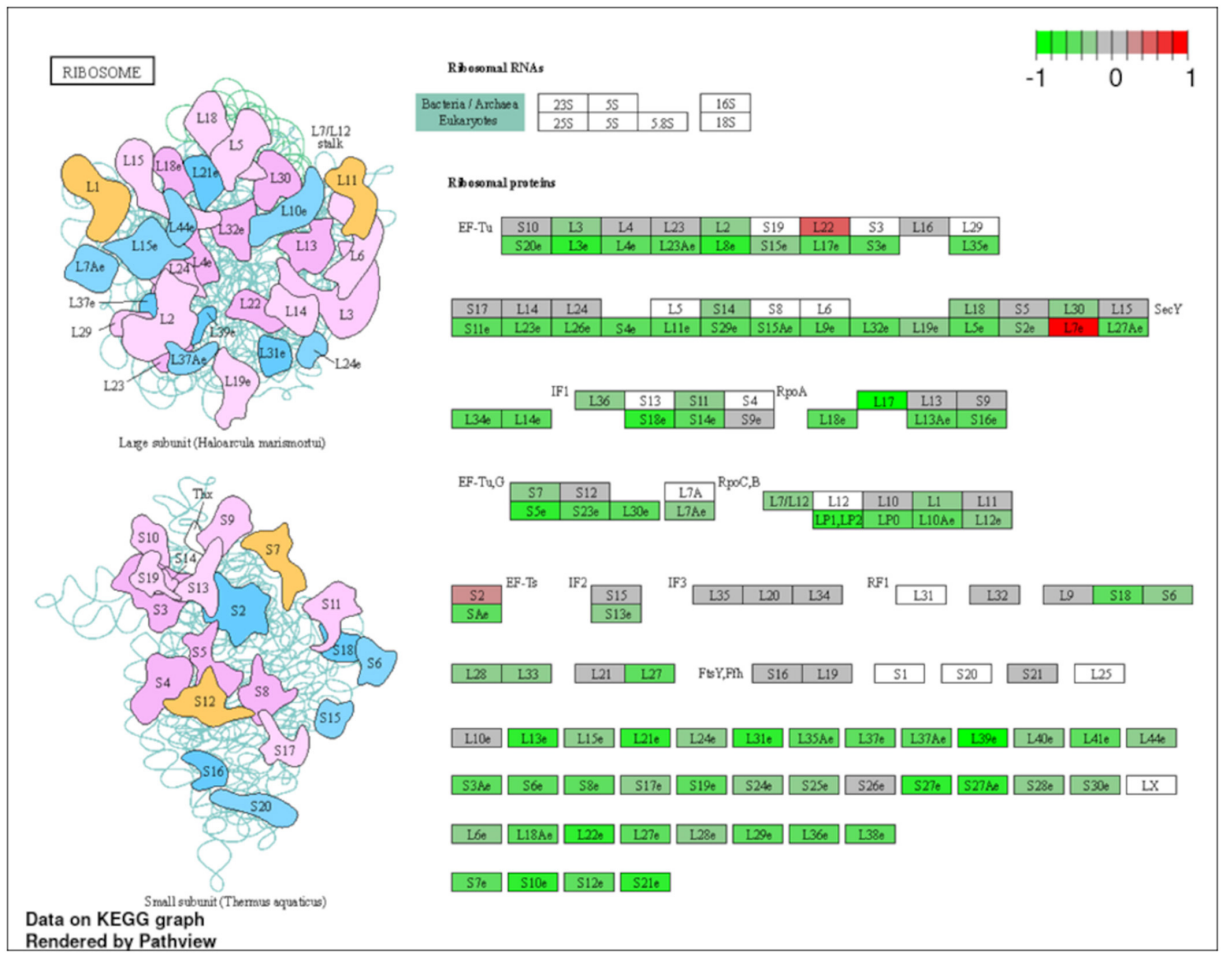
Genes involved in ribosomal pathway in pulmonary sarcoidosis PBMCs by KEGG. A graphical illustration of KEGG pathway analysis of DEGs (log_2_-fold change with FDR < 0.05) related to ribosomal signaling pathway in pulmonary sarcoidosis PBMCs.

To explore the pathways that are modulated in PBMCs of pulmonary sarcoidosis compared to that of healthy controls, we performed enrichment analysis (GO analysis and KEGG pathway analysis) using GSEA (FDR < 0.05, and fold-change > 2 or < 0.5). As shown in Table 2A, GO analysis revealed that various biological processes were enriched, such as “Cellular response to interleukin-1 (IL-1)” “Cellular response to IFN-γ,” “Positive regulation of interleukin-6 (IL-6) production,” “interleukin-8 (IL-8) secretion,” “Mononuclear cell migration,” “Regulation of mononuclear cell migration” and “response to lipopolysaccharide.” As shown in Table 2B, KEGG pathway analysis revealed that multiple pathways that include “TNF signaling pathways,” “IL-17 signaling pathways,” and “Toll-like receptor signaling pathways,” “Phagosome” and “Ribosome” were enriched; the majority of the genes involved in TNF, IL-17, and Toll-like receptor signaling pathways, and phagosome were upregulated (Figures 4–7), while most genes involved in ribosome pathway were downregulated (Figure 8).

**Table 2:** Enrichment analysis of transcriptomic data (pulmonary sarcoidosis vs. healthy controls).

**A. Gene ontology (biological process): relevant terms were excerpted**	
**Term (gene ontology: biological process) with upregulated genes**	***P*** **-value**
Cellular response to interleukin-1	1e-03
Response to lipopolysaccharide	1e-03
Cellular response to interferon-gamma	1e-03
Regulation of interleukin-1 beta production	1e-03
Positive regulation of interleukin-6 production	1e-03
Interleukin-8 secretion	1e-03
Mononuclear cell migration	1e-03
Regulation of mononuclear cell migration	1e-03
**B. KEGG pathway: relevant terms were excerpted**
**Term (KEGG pathway) with upregulated genes**	***P*** **-value**
TNF signaling pathway	5.6e-04
IL-17 signaling pathway	5.6e-04
Toll-like receptor signaling pathway	5.6e-04
Phagosome	5.6e-04
**Term (KEGG pathway) with downregulated genes**	***P*** **-value**
Ribosome	1.2e-03

Significant difference in mean age between sarcoidosis and healthy controls was detected (Table 1). Age is a very important factor that could influence the transcriptional signatures, therefore, additional transcriptome analysis was performed focusing on age by comparing the data of younger age (*n* = 4) with those of older age (*n* = 4) (mean age: 49 ± 4.6 vs. 67 ± 6.5, *p* < 0.05) to see if the findings described above become biologically irrelevant. PCA was performed by comparing gene expression patterns between older and younger patients with pulmonary sarcoidosis. The results showed that the two groups appeared to be undifferentiated, as shown in Figure S1. Of the 18,139 genes expressed in these samples, only two DEGs was detected (FDR < 0.05 and fold-change > 2 or < 0.5) and no term was detected by enrichment analysis (GO analysis and KEGG pathway analysis).

Sex difference is also a very important factor that could influence the transcriptional signatures. Therefore, additional transcriptome analysis was performed focusing on gender by comparing the data of females (*n* = 5) with those of males (*n* = 3) to see if the findings described above become biologically irrelevant. PCA was performed by comparing gene expression patterns between the male and female patients with pulmonary sarcoidosis. The results showed that the two groups appeared to be divided, as shown in Figure S2. Of the 18,139 genes expressed in these samples, only 14 DEGs were detected (FDR < 0.05 and fold-change > 2 or < 0.5) and terms of “Protein demethylation” and “Oxygen transport” was enriched by GO analysis.

Multiorgan involvements were observed in 3 out of 8 pulmonary sarcoidosis patients (Table 1), therefore, additional transcriptome analysis was performed by comparing data of patients with multiorgan involvements (*n* = 3) with those having only pulmonary lesion (*n* = 5) to clarify if differences in transcriptome of PBMCs exist between them. The results of PCA showed that the two groups appeared to be undifferentiated, as shown in Figure S3. Next, we compared the DEGs in PBMCs between the two groups. Of the 18,139 genes expressed in these samples, only five DEGs was detected as downregulated (FDR < 0.05 and fold-change > 2 or < 0.5) and terms of “Antibiotic catabolic process” and “Oxygen transport” were enriched by GO analysis.

## Discussion

In the present study, transcriptome analysis indicated that gene expression profiles differ in PBMC level between pulmonary sarcoidosis and healthy controls, probably reflecting the pathobiological state with granuloma formation. To our knowledge, the present study revealed, for the first time, the potential difference in gene expression profiles of PBMCs between healthy controls and pulmonary sarcoidosis without the influence of any immunomodulatory drugs such as steroids and/or immunosuppressant drugs. The present gene expression study using heterogeneous cell mixtures lacks insight into cell type-specific immune dysregulation. However, bulk RNA evaluation at the PBMC level could reflect some aspects of the clinical phenotype as a final product of cell-cell interactions among blood immune cells. Of note, changes in transcriptome of lots of mediators and pathways that were reported to be related to sarcoidosis, were detected in PBMC levels.

The present study revealed that 270 genes were differentially expressed in PBMCs between subjects with pulmonary sarcoidosis and healthy controls. Enrichment analysis with GO analysis revealed that various biological pathways were enriched such as “Cellular response to IL-1,” “Cellular response to IFN-γ,” “Positive regulation of IL-6,” “IL-8 secretion,” “Mononuclear cell migration,” “Regulation of mononuclear cell migration,” and “Response to lipopolysaccharide (LPS),” all of which are reported to be in relation to granuloma formation in sarcoidosis lesion (Table 2A). In addition, enrichment analysis with KEGG pathway analysis showed that genes involved in TNF, IL-17 and toll-like receptor signaling, and phagosomes were mostly upregulated, while ribosome-related genes were mostly downregulated (Table 2B). The terms that were led by enrichment analysis in the present study were not described in a previous study that performed transcriptome analysis with PBMC to compare healthy controls with pulmonary sarcoidosis patients (10). One of the reasons could be that some transcriptome data of sarcoidosis patients were from those who received steroids and/or immunosuppressant drugs (10). Therefore, the results of the present study could provide notable new insights into the sarcoidosis research field as transcriptome analysis was performed with DEGs in sarcoidosis patients who did not receive anti-inflammatory drugs such as steroids and/or immunosuppressant drugs. Our results also suggest that steroids or immunosuppressant drugs may certainly suppress the pro-inflammatory signals at PBMC level in pulmonary sarcoidosis.

First, the terms “Cellular response to IL-1,” and “TNF signaling pathway” were listed in this study (Table 2; Figure 4). In sarcoidosis, unstimulated sarcoid-derived alveolar macrophages have been shown to intrinsically produce increased amounts of IL-1 and TNF-α (13, 14). IL-1 is a cytokine with potent pro-inflammatory properties implicated in the pathogenesis of sarcoidosis (15). TNF has also been shown to play a pivotal role in orchestrating innate inflammatory responses, leading to granuloma formation (16). Together, the results of the present study support those of previous studies that indicate a central role of IL-1 and TNF signaling in sarcoidosis.

Next, “IL-17 signaling pathway” was listed in this study (Table 2B; Figure 5). IL-17 has been designated as IL-17A, a founding member of the IL-17 cytokine family, which are produced by human Th17 cells. IL-17 induces the mobilization, recruitment, and activation of neutrophils and triggers the production of proinflammatory cytokines and chemokines by a broad range of cellular targets, including epithelial cells, endothelial cells, and macrophages (17–19). Regarding IL-17 in sarcoidosis, it was reported that the primary Th17-cytokine, IL-17A, is an important mediator of granuloma formation and maturation (20). In addition, it was also reported that IL-17A could potentiate IFN-γ-induced giant cell formation (21), and can stimulate TNF-α production by macrophages (22). The results of the current study support previous reports that suggest a central role for IL-17 signaling in sarcoidosis.

The terms “Response to lipopolysaccharide” and “Toll-like signaling pathway” were listed in this study (Tables 2A,B; Figure 6). LPS is the major outer surface membrane component present in almost all gram-negative bacteria and acts as an extremely strong stimulator of innate or natural immunity in diverse eukaryotic species ranging from insects to humans. Sarcoidosis is generally thought to occur when genetically susceptible individuals are exposed to disease-related antigens, triggering a Th1-dominant immune response that eventually leads to the formation of granulomas. LPS has been identified in the lungs of sarcoidosis patients, and PBMCs of sarcoidosis patients show hypersensitivity reactions when stimulated with LPS (23). Furthermore, LPS is known to induce a Th1-based immune response that promotes IFN-γ, a key cytokine involved in granuloma formation in sarcoidosis (24, 25). The present study showed upregulation of many genes related to the “Toll-like receptor signaling pathway,” indicating that altered gene expression is important in the activation of pattern recognition receptors and signaling molecules (Figure 6). Granulomatous inflammation is thought to be a dysregulated antigenic response to unknown environmental exposure in genetically susceptible individuals (26). Together, the present study supports previous reports that suggest possible major mechanistic roles for the innate immune signaling pathway in granuloma formation.

Next, the terms “Cellular response to IFN-γ,” “Cellular response to IL-6,” and “IL-8 secretion” were listed in the GO analysis (Table 2A). IFN-γ was originally identified as a “macrophage-activating factor,” and macrophages are a major physiological target for IFN-γ action (27). The activation of macrophages in sarcoid lesions is thought to be driven by Th1 cell immune responses and mediated by several cytokines, including IFN-γ. IFN-γ is a cytokine that is primarily produced by cells of the immune system, including innate-like lymphocyte populations, such as natural killer (NK) cells and innate lymphoid cells (ILCs), and adaptive immune cells, such as Th1 cells and CD8^+^ cytotoxic T cells (28). IFN-γ is also derived from Th17.1 cells, which may have an important role in sarcoidosis (4). IL-6 induces the development of Th17 cells from naïve T cells together with transforming growth factor β (TGF-β) and inhibits anti-inflammatory regulatory T cells (29). IL-8 is a potent chemokine for neutrophils and is also chemotactic for T cells (30), and previous report suggests that this chemokine might be involved in delayed hypersensitivity reactions (31). Together, the results of the present study support previous studies that suggest possible central roles for IFN-γ, IL-6, and IL-8 in sarcoidosis.

The terms “mononuclear cell migration” and “regulation of mononuclear cell migration” were listed in the GO analysis (Table 2A). “Mononuclear cell migration” or “regulation of mononuclear cell migration” refers to the action of mononuclear cells moving to the tissues and organs that require functions of the immune system. In sarcoidosis, Garman et al. reported altered expression of genes important for activation (pattern recognition receptors and signaling molecules) and migration (adhesion molecules and chemokine receptors) in monocytes isolated from sarcoidosis patients compared to those from controls (32). Cells in the peripheral blood are thought to be directly involved in the formation of granulomas in the lungs. Monocytes in the blood, along with antigen-presenting cells present in tissues such as dendritic cells and alveolar macrophages, act as sentinels to detect potential threats to the host, leaving the vascular space and patrolling in the interstitial spaces of the lung (33). The results of the present study support previous reports that indicate the importance of PBMC migration for granuloma formation in pulmonary sarcoidosis.

Next, the term “Phagosome” was listed in the KEGG pathway analysis (Table 2B; Figure 7). Phagocytosis is a cellular process that ingests and eliminates particles, including microorganisms, foreign substances, and apoptotic cells. It is an essential process in tissue homeostasis, and macrophages and monocytes are the dedicated cells for this process. The suggested upregulation in phagocytosis supports the notion that the development of lung sarcoidosis may be associated with the response to an unknown external exposure requiring antigen processing and presentation. Taken together, it would be reasonable to see that the phagosome-related genes were mostly upregulated at PBMC levels.

Finally, the term “Ribosome” was listed in the KEGG pathway analysis (Table 2B; Figure 8). Ribosome biogenesis is an extremely energetically expensive cellular process that has long been linked to human health and disease. Taljera et al. recently reported that RNA-seq analysis of monocytes from sarcoidosis patients showed that pathways associated with ribosomes were downregulated, similar to the results of the present study (34). Together, downregulation of ribosomal constituent proteins in PBMCs, including monocyte levels, could be related to the pathobiological state of sarcoidosis. However, it remains unclear how ribosomes are involved in the pathogenesis of sarcoidosis; therefore, further studies are needed to clarify the role of ribosomes in the etiology of pulmonary sarcoidosis.

The current study indicates functional roles of PBMCs, including monocytes, the so-called circulating precursors of macrophages, in the etiology and pathobiology of pulmonary sarcoidosis. However, this study had several limitations. First, this study was conducted in a single center in Japan, and the sample size was small, which potentially limited the generalizability of findings in the current study. Attention should also be paid to the fact that sarcoidosis group is very heterogeneous in terms of different stages at chest radiograph findings. The subjects' nationality would affect the genetic risk of developing sarcoidosis. Our previous study on assessing the incidence of concurrent sarcoid-like granuloma in patients with malignancy indicated that Japanese subjects were less likely to have stage 2, 3, and 4 sarcoidosis than Americans (OR 0.31, 95% CI 0.13–0.72, *p* = 0.007) and were less likely to develop sarcoid-like granulomas within 5 years of cancer diagnosis (OR 0.31, 95% CI 0.13–0.72, *p* = 0.006) (35). The ethnicity of Japanese patients, which may make it difficult for sarcoidosis granulomas to form, might influence the results of this study. It is well known that sarcoidosis is a polygenic, multifactorial disease (36). Many association studies have attempted to identify genetic susceptibility in sarcoidosis, and various genes have been found to increase the risk of developing sarcoidosis, including chemokine receptors, tumor necrosis factor (TNF)-α, and several HLA loci and MHC class II antigens (37). Therefore, careful interpretation of the transcriptional signature, as well as genetic linkage analysis, is required considering ethnicity. Second, this study shows the characteristics of PBMC gene expression at the time of diagnosis in patients with pulmonary sarcoidosis and was not intended to explore the relationship between transcriptome expression and prognosis of sarcoidosis. Third, we showed that gene expression of cytokines such as TNF and IL-17 was upregulated in patients with pulmonary sarcoidosis, but we did not confirm that by measuring interleukin levels in PBMCs or lung tissue. Therefore, further studies are needed to validate the mechanistic roles of PBMCs in sarcoidosis.

In the present study, additional transcriptome sub-analyses were performed to see apparent influence of differences in age, sex, and the lesions, although the sample number in each group was small (Figures S1–S3). The results showed that no same term as that shown in Tables 2A,B was detected in the three types of comparison by enrichment analysis (GO and KEGG pathway analysis), potentially implying that age and sex have no apparent influence on developing pulmonary sarcoidosis, and other than transcriptome signature in PBMCs may be more relevant to multiorgan involvement. However, it needs to be further evaluated with more samples to form a firm conclusion.

In conclusion, the current study suggests that bulk gene expression patterns in PBMCs are different between healthy controls and patients with pulmonary sarcoidosis. Most of the terms from enrichment analysis in the present study were different from those in a previous study that performed transcriptome analysis of PBMC comparing healthy controls with pulmonary sarcoidosis patients, more than half of whom received anti-inflammatory drugs. The changes in gene expression patterns of PBMCs could reflect the existence of sarcoidosis lesions and have an influence on granuloma formation in sarcoidosis. These new findings are important to strengthen our understanding of the etiology and pathobiology of sarcoidosis and indicate a potential therapeutic target for sarcoidosis.

## Data Availability Statement

The datasets presented in this study can be found in online repositories. The names of the repository/repositories and accession number(s) can be found below: https://www.ncbi.nlm.nih.gov/, GSE192829.

## Ethics Statement

The studies involving human participants were reviewed and approved by Graduate School of Medicine, Chiba University. The patients/participants provided their written informed consent to participate in this study.

## Author Contributions

TK and KT: conceptualization. KY, TK, DI, TI, and MA: data curation. KY, HS, TK, YH, and OO: formal analysis and methodology. KT: funding acquisition. KY, HS, TK, and KT: investigation. TK, KT, and TS: project administration. OO, KT, and TS: supervision. KY and HS: visualization. KY and TK: writing – original draft. TK, OO, KT, and TS: writing – review and editing. All authors contributed to the article and approved the submitted version.

## Funding

This research was funded by a research grant from the Intractable Respiratory Diseases and Pulmonary Hypertension Research Group, the Ministry of Health, Labour and Welfare, Japan (Grant Number 20FC1027).

## Conflict of Interest

The authors declare that the research was conducted in the absence of any commercial or financial relationships that could be construed as a potential conflict of interest.

## Publisher's Note

All claims expressed in this article are solely those of the authors and do not necessarily represent those of their affiliated organizations, or those of the publisher, the editors and the reviewers. Any product that may be evaluated in this article, or claim that may be made by its manufacturer, is not guaranteed or endorsed by the publisher.

## Supplementary Material

Figure S1 Principal component analysis (PCA). Principal component analysis (PCA) shows that two groups in pulmonary sarcoidosis are undifferentiated between older patients and younger patients.

Figure S2 Principal component analysis (PCA). Principal component analysis (PCA) shows that two groups in pulmonary sarcoidosis appeared to be differentiated between female and male.

Figure S3 Principal component analysis (PCA). Principal component analysis (PCA) shows that two groups in pulmonary sarcoidosis are undifferentiated between patients with multiorgan involvement and those with only pulmonary lesions.
