# Supplementary figures and images for: Transcriptome Analysis of Peripheral Blood Mononuclear Cells in Pulmonary Sarcoidosis
Source: Front Med (Lausanne). 2022 Jan 24;9:822094. doi: 10.3389/fmed.2022.822094 (PMC8818883; doi:10.3389/fmed.2022.822094)

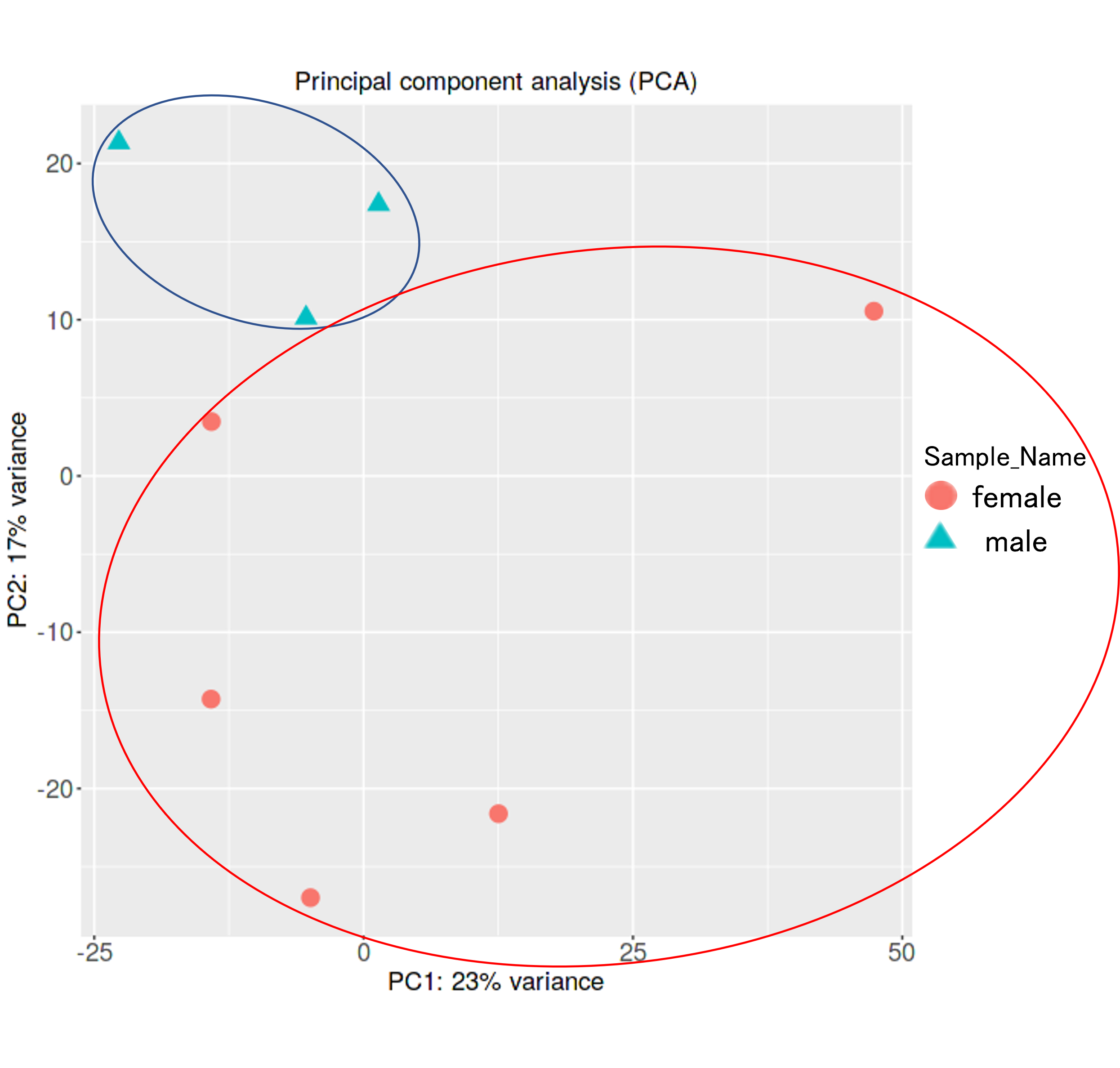

Supplement: Figure S1 — Principal component analysis (PCA). Principal component analysis (PCA) shows that two groups in pulmonary sarcoidosis are undifferentiated between older patients and younger patients. [file Image_1.TIF]

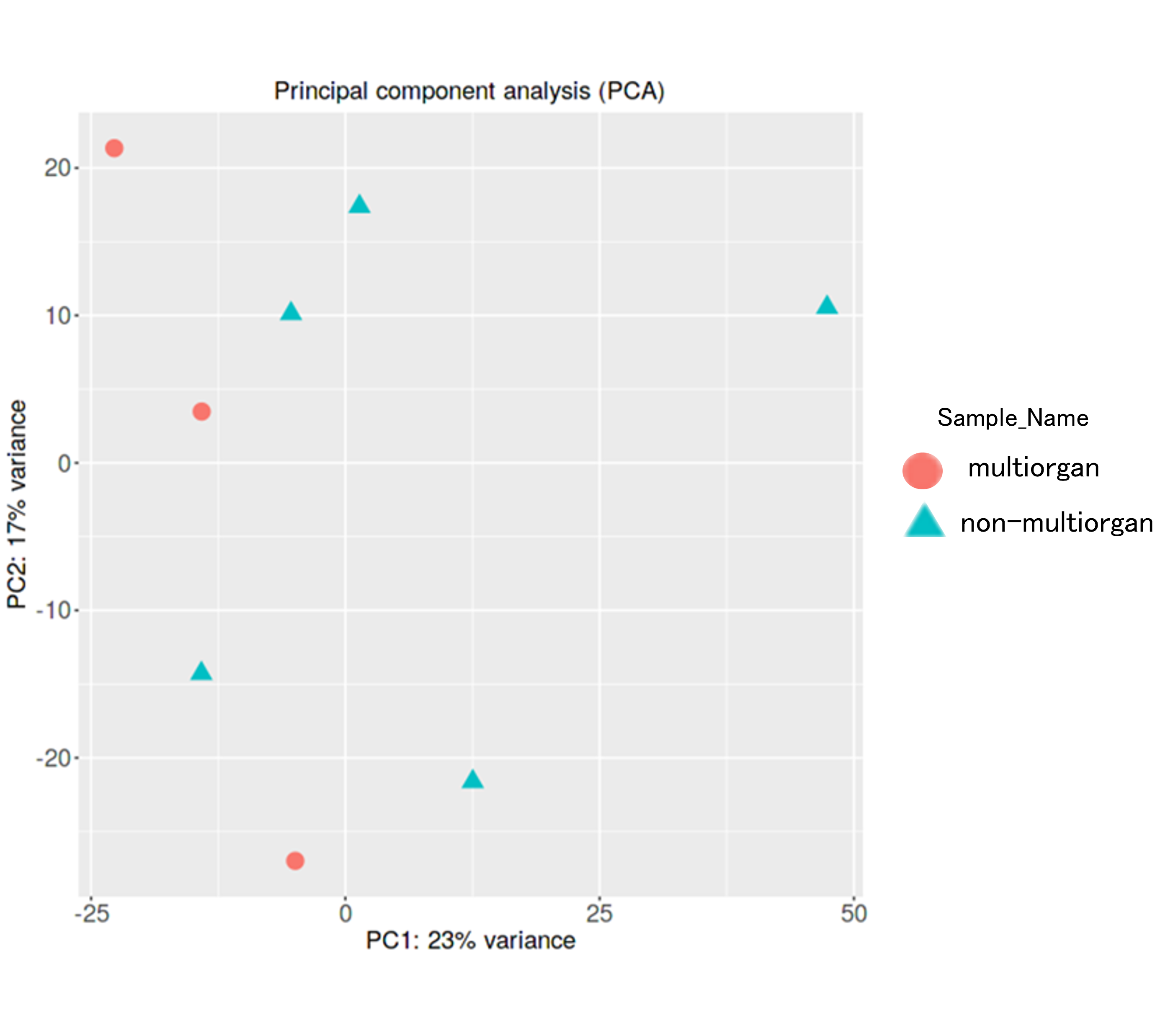

Supplement: Figure S2 — Principal component analysis (PCA). Principal component analysis (PCA) shows that two groups in pulmonary sarcoidosis appeared to be differentiated between female and male. [file Image_2.TIF]

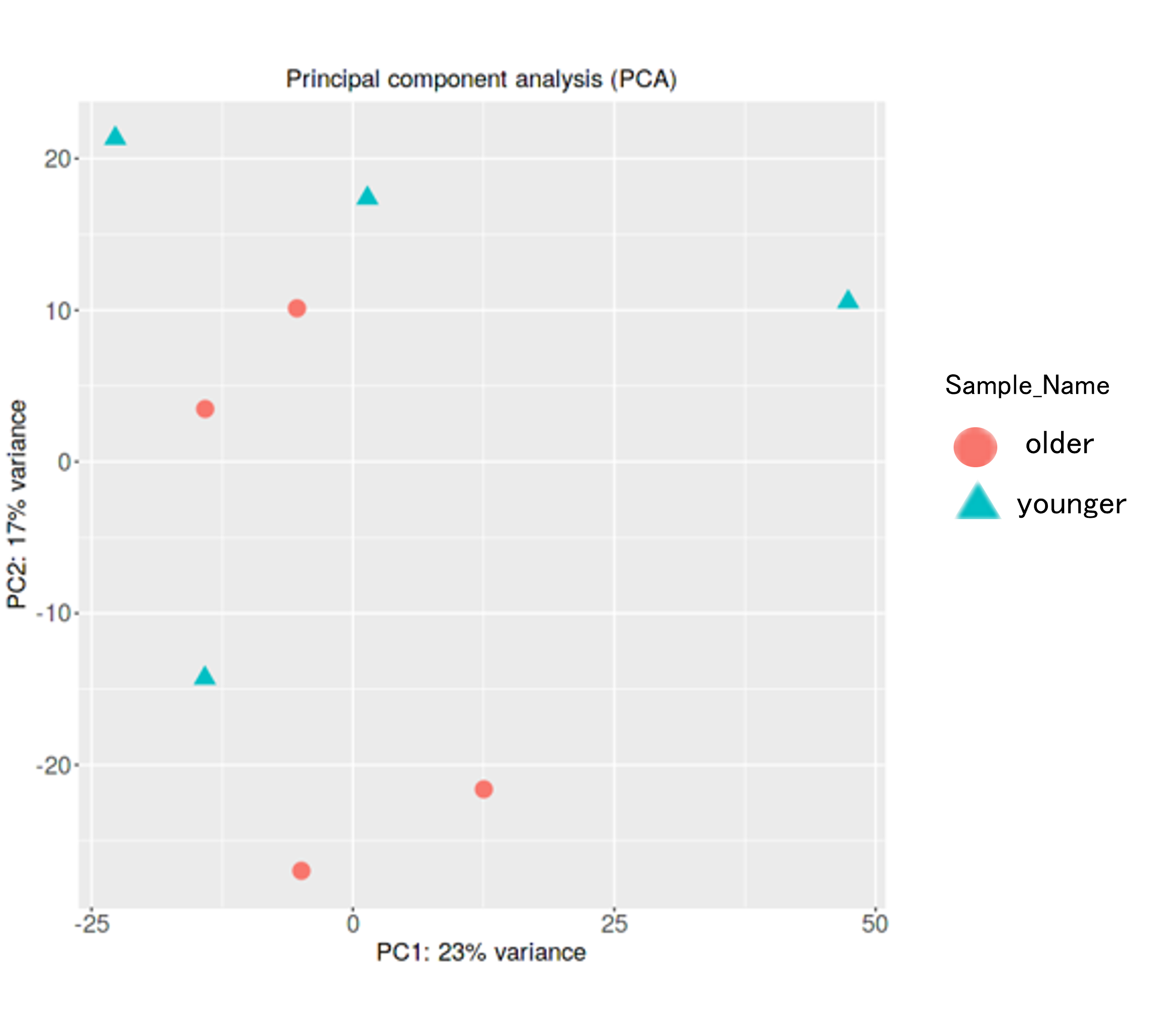

Supplement: Figure S3 — Principal component analysis (PCA). Principal component analysis (PCA) shows that two groups in pulmonary sarcoidosis are undifferentiated between patients with multiorgan involvement and those with only pulmonary lesions. [file Image_3.TIF]
